# Insights into the Structure, Metabolism, Biological Functions and Molecular Mechanisms of Sialic Acid: A Review

**DOI:** 10.3390/foods13010145

**Published:** 2023-12-31

**Authors:** Dan Li, Qinlu Lin, Feijun Luo, Hanqing Wang

**Affiliations:** 1 Hunan Key Laboratory of Grain-Oil Deep Process and Quality Control, Hunan Key Laboratory of Processed Food for Special Medical Purpose, National Engineering Laboratory for Deep Process of Rice and Byproducts, College of Food Science and Engineering, Central South University of Forestry and Technology, Changsha 410004, China; ld1764409498@163.com; 2 Hunan Engineering Research Center of Full Life-Cycle Energy-Efficient Buildings and Environmental Health, School of Civil Engineering, Central South University of Forestry and Technology, Changsha 410004, China

**Keywords:** sialic acid, biological functions, molecular mechanisms, gut microbiome

## Abstract

Sialic acid (SA) is a kind of functional monosaccharide which exists widely in edible bird’s nest (EBN), milk, meat, mucous membrane surface, etc. SA is an important functional component in promoting brain development, anti-oxidation, anti-inflammation, anti-virus, anti-tumor and immune regulation. The intestinal mucosa covers the microbial community that has a significant impact on health. In the gut, SA can also regulate gut microbiota and metabolites, participating in different biological functions. The structure, source and physiological functions of SA were reviewed in this paper. The biological functions of SA through regulating key signaling pathways and target genes were discussed. In summary, SA can modulate gut microbiota and metabolites, which affect gene expressions and exert its biological activities. It is helpful to provide scientific reference for the further investigation of SA in the functional foods.

## 1. Introduction

Sialic acid (SA), a subset of α-keto aldonic acids, is found in a variety of foods, animals and organisms [1]. SA is an acidic amino sugar with a nine-carbon skeleton. SA has different forms, usually including glycolipids or glycoproteins. The structure of SA usually consists of glucan and SA and forms with different SA bonds. Its structural diversity leads to many biological functions, including antiviral activity [2], antioxidant activity [3], anti-adhesion activity [4], anti-inflammatory property [5], and improving learning and memory [6], promoting bone growth and prebiotics [7,8]. SA can participate in important pathological and physiological processes and can be used as nutrition, drugs and drug carriers to prevent diseases [1]. Therefore, SA is a promising active substance, which can improve human health.

The intestinal mucosa covers a wide range of microbial communities that have a profound impact on health. The intestinal mucus layer is a physical barrier to promote intestinal homeostasis. Mucin is the main component of mucus and the main source of SA. SA on the mucosal surfaces comes from the diet and can be metabolically integrated into the host surface glycans. SA is located at the end of the mucin glycan and can directly contact with gut microbes. Some studies indicate that SA is extensively modified in the gut environment and plays an important functional role in host-microbe interactions [9,10]. In recent years, many studies have been carried on the health benefits and mechanism of SA. The results show that SA can exert its biological functions by affecting the gut microbiota and metabolites, regulating signaling pathways and key target gene expressions, which attracts more and more researchers’ attention.

This paper systemically summarized the structure, physical and chemical characteristics, biological functions of SA, especially SA regulates gut microbiota to exert different biological functions and mechanisms. It will provide reference for further study of SA in the field and also promote the development of functional foods.

## 2. Main Structures, Distributions and Metabolism of SA

### 2.1. Structures of SA

**Figure 1:**
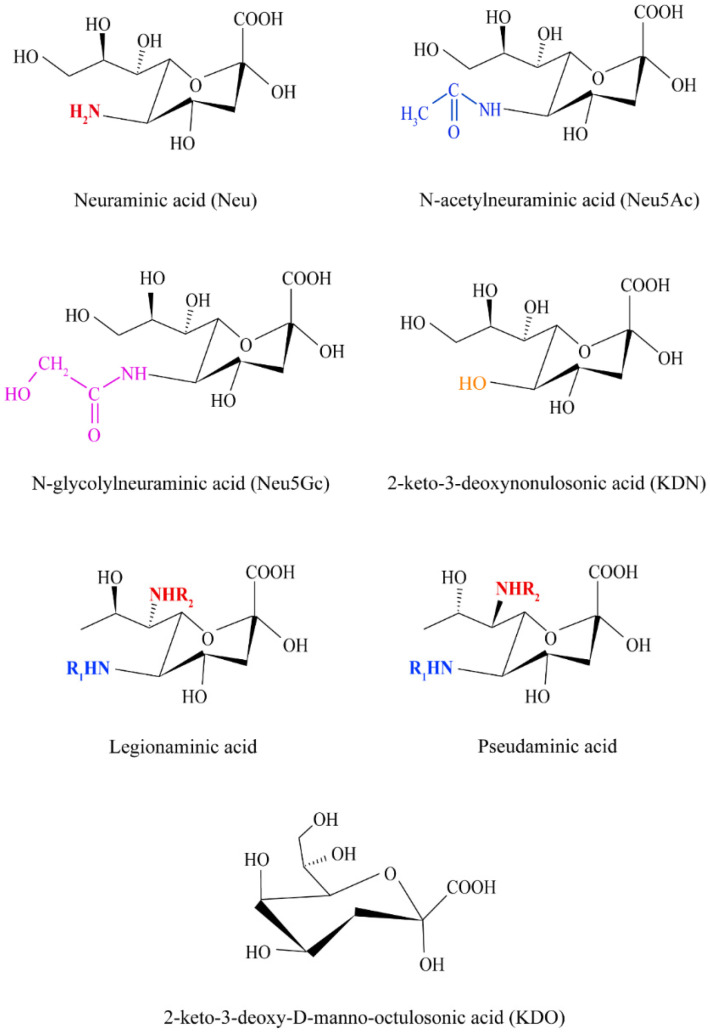
Main structures of sialic acid.

SA is often found in the outer layer of cell membrane or released by mild acid hydrolysis of glycoconjugates. It is a nine-carbon oligosaccharide with a negative charge derived from neuraminic acid (Neu). More than 50 derivatives of SA have been reported. SA is replaced by O-methylation, O-sulfation, O-acetylation or phosphorylation at different positions, resulting in structural diversity. The major types of changes at C5 position are N-acetylneuraminic acid (Neu5Ac), N-glycolylneuraminic acid (Neu5Gc), deaminated neuraminic acid (KDN), and Neu in mammals. The main forms of SA are Neu5Ac and Neu5Gc in humans. Neu5Gc has a glycolyl group at C5-position and Neu5Ac with N-acetyl at C5-position. Except for cancers, mutations in the cytidine monophosphate N-acetylneuraminic acid hydroxylase (CMAH) gene prevent the body from synthesizing Neu5Gc in vivo [11]. However, Neu5Gc can be obtained from dietary sources. Neu5Ac is the most common member of SA. Besides, a set of nonulosonic acids that share the nine-carbon backbone of SA are found in prokaryotes. Legionaminic acid (5,7-diamino-3,5,7,9-tetradeoxy-D-glycero-D-galacto-nonulosonic acid) and pseudaminic acid (5,7-diamino-3,5,7,9-tetradeoxy-L-glycero-L-manno-nonulosonic acid) have similar structures, and they are common nonulosonic acids. KDO (2-keto-3-deoxy-D-manno-octulosonic acid) is an eight-carbon α-keto ulosonic acid, which shares similarities to KDN (see Figure 1).

**Figure 2:**
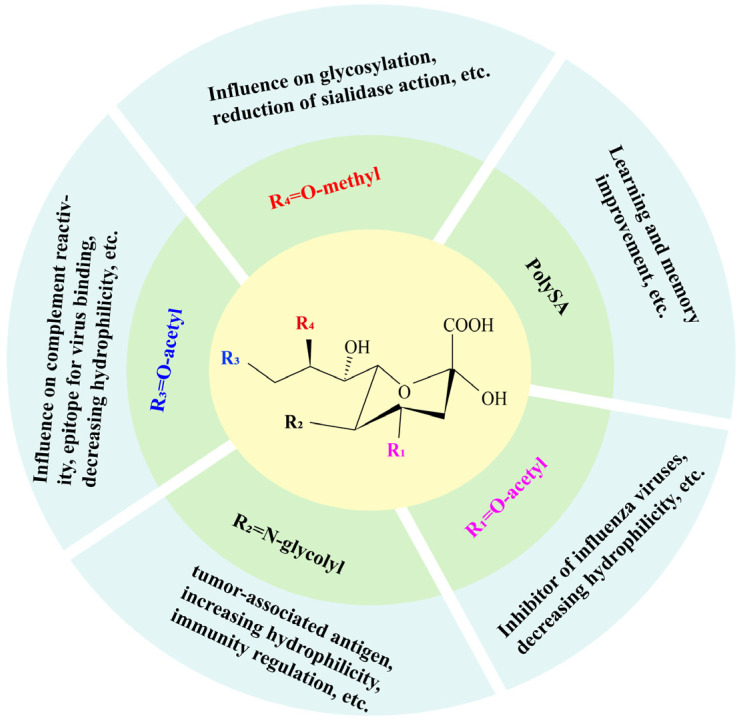
Main physiological functions of the modified sialic acid.

Small amounts of SA are present in free form, most of which bind to oligosaccharides, lipids, or proteins [12]. SA is a functional residue at the end of oligosaccharide chain in the cell membrane, which regulates many physiological processes [13]. SA can be modified to galactose and N-acetyl-galactosamine through α-(2→3) or α-(2→6) glycosidic bond. Polysialic acid (PolySA), mainly attached to the neural cell adhesion molecule (NCAM), is formed by the polymerization of SA molecule via α-(2→8) and/or α-(2→9) glycosidic linkage, which usually occurs in the brain gangliosides [14]. Because of the diversity of SA modifications [15], more and more attention has been paid to the relationship between structure and function of SA (see Figure 2).

### 2.2. Distributions of SA

The distribution of SA in nature is extensive, and it is usually presented in conjugated forms. SA exists widely in many animals (i.e., mammals, reptiles, birds, etc.), some viruses, and bacteria [16]. It exists in liver, lung, serum, mucus substance and body fluid of higher animals and the content of SA in human brain is also higher than that in other organs. The major forms of SA, including gangliosides, glycoproteins, and free SA, play important roles in neurotransmission, differentiation, and synaptogenesis [17,18]. SA can be obtained from foods that include red meat, fish, milk and milk-based products, casein, eggs, edible bird’s nest (EBN) and breast milk [19,20]. About 73% SA in human milk binds to oligosaccharides to form sialyloligosaccharides. SA is an important component of EBN, accounting for about 10% of the total weight, and the content of SA is also the most important quality index of EBN [21]. According to publications, the contents of SA in EBN are ranging from 5.86 ± 0.39 g/100 g to 19.29 ± 0.31 g/100 g [22]. Some animal foods are the main source of Neu5Gc, such as beef, which contains Neu5Gc up to 3.6 ± 0.5 mg/100 g [23]. Overall, animal foods are a major source of SA, and vegetarians may need to supplement SA from other sources.

### 2.3. Metabolism of SA

Diet is a rich source of exogenous SA, such as EBN, breast milk, dairy products, eggs, etc. The SA glycoconjugates in food are decomposed into SA, amino acids, fatty acids and other substances in the intestine, and then SA monomers and synthesized PolySA modulate physiological processes after cellular uptake, such as immune cell and nerve cell [24]. The biological functions of SA are closely related with its metabolism.

**Figure 3:**
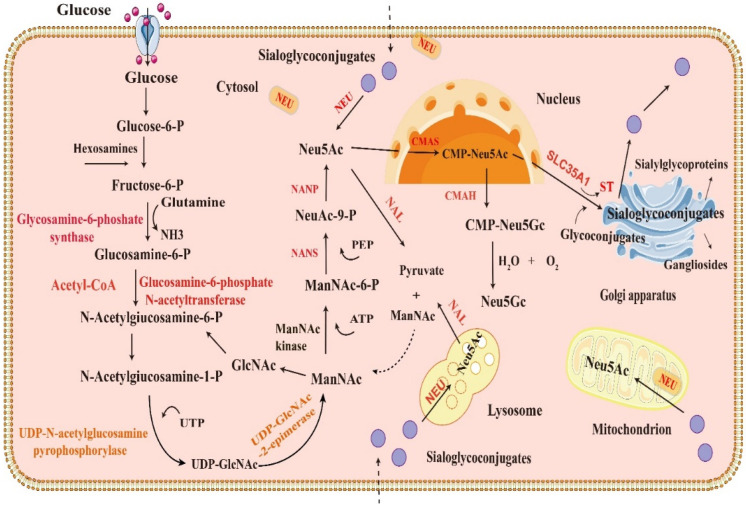
Process diagram of sialic acid metabolism in mammalian cells. Biosynthesis, transformation, transfer and degradation of sialic acid during its metabolism in mammalian cells.

In addition to the exogenous supplement of SA, it can also be synthesized endogenously and the major SA is located in the cytosol, lysosome and mitochondrion (see Figure 3). In mammalian cells, SA biosynthesis mainly starts with glucose or other product of glycolysis in the cytosol. It binds to glutamine, acetyl coenzyme A (Acetyl-CoA) and UTP to generate uridine diphosphate-*N*-acetylglucosamine (UDP-GlcNAc). UDP-GlcNAc is catalyzed by UDP-GlcNAc-2-epimerase and produces N-acetylmannosamine (ManNAc), and then ManNAc is converted to ManNAc-6-phosphate (ManNAc-6-P) by ManNAc kinase [25], followed by condensation with phosphoenolpyruvate (PEP) and N-acetylneuraminate-9-phosphate synthase (NANS) to produce N-acetylneuraminic-9-phosphate (NeuAc-9-P) [26]. NeuAc-9-P can be catalyzed by N-acetylneuraminate-9-phosphate phosphatase (NANP) and is dephosphorylated to produce Neu5Ac [27]. Finally, Neu5Ac can be catalyzed by cytosine 5′-monophosphate N-acetylneuraminate synthetase (CMAS) and produce cytidine 5′-monophosphate-N-acetylneuraminic acid (CMP-Neu5Ac) in the nucleus [28]. In most mammals, CMP-Neu5Ac is catalyzed by CMAH and produce CMP-Neu5Gc in the cytosol. However, the human body can not synthesize CMP-Neu5Gc due to the lack of CMAH. CMP-Neu5Ac can be transported to the Golgi apparatus by the transporter (SLC35A1) and activated by sialyltransferases, thereby forming sialoglycoconjugates such as sialylglycoproteins and gangliosides, and the sialoglycoconjugates are to the cell membrane or extracellular secretion (see Figure 3).

Meanwhile, the sialoglycoconjugates can be hydrolyzed by neuraminidase (NEU) to form Neu5Ac. The released Neu5Ac enters into another cycle of sialoglycoconjugates in the cytosol. The main distributions of neuraminidases are the cytosol [29], lysosomal [30], mitochondria [31], plasma membrane [32], etc. Due to the lack of the specific transporter on the cell membrane, extracellular SA enters into the cell by endocytosis or pinocytosis [33]. They can directly enter into the formation cycle of sialoglycoconjugates. Moreover, Neu5Ac can also be catalyzed by N-acetylneuraminate lyase (NAL) and are degraded to ManNAc and pyruvate [34]. ManNAc is involved in another cycle of SA biosynthesis. The normal range of total SA is 1.58–2.22 mmol/L in human plasma and more than 70% of SA are glycoprotein, and the content of free SA is about 0.5–3 μmol/L. The normal range of human serum lipoprotein SA is 10–50 μmol/L [35]. The SA synthesis mainly occurs in the liver, and the immature liver of newborn can’t address the needs by metabolism. For the human, especially the infants, exogenous supplementation of SA is required.

## 3. Biological Functions and Molecular Mechanisms of SA

**Figure 4:**
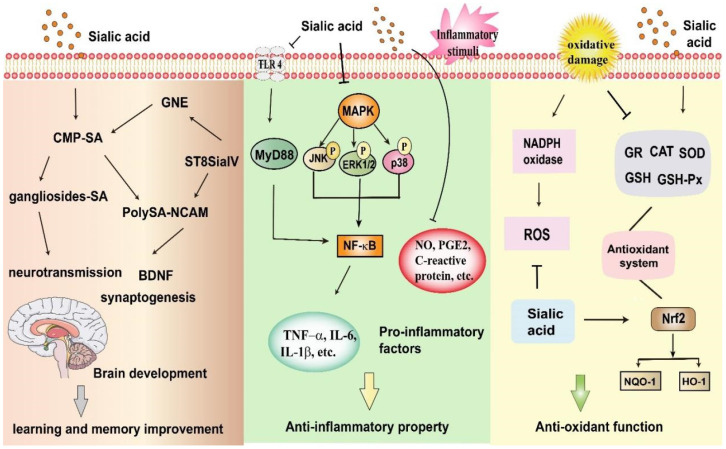
Main molecular mechanisms regulated by sialic acid. Brain development increases the need for sialic acid (SA). GNE (UDP-N-acetyl-glucosamine-2-epimerase/N-acetylmannosamine kinase) is a critical enzyme for SA biosynthesis. SA is activated to form CMP-SA in the nucleus. Then CMP-SA is transported into the Golgi apparatus and serves as the substrate of SA for the sialyltransferases, like the polysialyltransferase-2,8-sialyltransferase IV and ST8SiaII. SA residues from CMP-SA are transferred to gangliosides and the neural cell adhesion molecule (NCAM), leading to the formation of polysialic acid on NCAM (PolySA-NACM). These sialylated structures play a central role in the neurotransmission and synaptogenesis, promoting the brain development. Under these conditions, learning and memory improvement are related to the upregulation of these gene expressions. SA also markedly reduces the inflammatory response. It is worked by the regulation of several signaling pathways, such as mitogen-activated protein kinase (MAPK) signal, nuclear factor-kappa B (NF-κB) pathway, and toll-like receptor 4 (TLR4) signaling pathway. SA effectively protects against oxidative damage. The levels of glutathione reductase (GR), catalase (CAT), superoxide dismutase (SOD), glutathione (GSH) and glutathione peroxidase (GSH-Px) were increased. The generations of reactive oxygen species (ROS) were reduced. Anti-oxidant function of SA is regulated by Nrf2 pathway, enhancing the expressions of heme oxygenase-1 (HO-1) and NADPH quinone oxidoreductase 1 (NQO-1).

SA has many biological functions, which are closely related to its regulation of signaling pathways and the expressions of some key target genes. In this paper, the different biological functions of SA and its molecular mechanisms are reviewed systematically in the following parts. The biological functions of SA in regulating signaling pathways and target genes are shown in Figure 4, and some details are also listed in Table 1.

### 3.1. Brain Development and Learning Behavior

The ganglioside is mainly in the cerebral cortex. SA significantly promotes ganglioside sialylation and plays a key role in synaptic connections and neural circuits. High concentration of SA is the integral structure and functional part of PolySA on NCAM, which plays an important role in promoting brain development and learning behavior [14]. Compared with formula-fed infants, breast-fed infants had significantly higher SA concentrations in the frontal cortex of the brain, which resulted in neurological and intellectual advantages [13]. It suggests that SA is an essential nutrient for synaptogenesis and neurodevelopment. Compared to control group, rats given oral SA during lactation generally showed better behavioral evaluation and enhanced long-term potentiation [36]. Human milk oligosaccharides (HMOs) are the main source of SA in the breast milk and can promote the neonatal growth and development. Sialylated HMOs, in the form of 3′sialylactose (3′SL) and 6′sialylactose (6′SL), account for the majority of SA in the breast milk. 6’SL deprivation in lactation impaired cognitive function in mice, while expressions of genes regulating neuronal formation, such as ganglioside or PolySA-NCAM, was also reduced in prefrontal cortex [37]. During early development, piglets were fed formula containing different levels of SA, and there was a dose-dependent relationship between ganglioside concentration in brain and SA content in feed. Importantly, there was a clear dose-response relationship between SA supplementation and mRNA expression levels of two learning-related genes, namely, UDP-N-acetyl-glucosamine-2-epimerase (GNE) and α-2,8-sialyltransferase IV (ST8SiaIV) [38]. Compared to control group, a higher percentage of pigs maintained cognitive function after 19 days of milk oligosaccharides with sialyllactose feeding. At the same time, the expressions of gene modifying SA metabolism, myelination and ganglioside biosynthesis were up-regulated by 1.2–1.5 folds in hippocampus [39]. Consistent with the positive role of SA in brain development, treatment with SA in the neonatal period had lasting cognitive improvements in adulthood. At the 60th day, the cognitive function of the corresponding adult mice was improved after injection of 240 mg/kg/day SA for 2 weeks [40].

Many studies demonstrated that exogenous administration of Neu5Ac in pregnant rats could effectively increase the brain Neu5Ac level of offspring and showed better learning and memory ability [41]. The maternal mice supplemented with 10 mg/kg EBN (rich in SA) for 6 weeks, the cognitive and neurological development of their first (F1)-generation and second (F2)-generation were promoted. EBN supplementation could increase the mRNA levels of GNE, ST8SiaIV, brain-derived neurotrophic factor (BDNF), and solute carrier family 17 member 5 (SLC17A5) in their offspring mice [42]. Xie et al. [43] observed that maternal mice supplemented with EBN during the pregnancy or lactation period facilitated learning and memory performances in the offsprings, which was attributed to the fact that EBN supplementation increased BDNF levels, the activities of superoxide dismutase (SOD) and choline acetyltransferase (ChAT), as well as the content of malondialdehyde (MDA) and the activity of acetylcholinesterase (AChE) were decreased in brain. These findings suggest that SA supplementation can enhance the learning and memory abilities via the regulation of related gene expressions.

### 3.2. Anti-Viral Capacity

SA plays an important role in the anti-virus action. The cell membrane is the decisive entry of the virus, and the cell receptor is very sensitive to the invasion of the virus. SA can interact with influenza viruses, thus interfering with the ability of viruses to bind to cells. It is a natural receptor for influenza viruses and an ideal target for anti-viral therapy. Respiratory viral infection is of great concern because of its high infection rate. SA is an important attachment site of viral protein in respiratory tract infection. Targeting SA on the surface of host cells may be suitable for inhibiting respiratory viral infection [56]. Severe acute respiratory syndrome coronavirus-2 (SARS-CoV-2) can be bound to SA on the host cell surface through spike protein, resulting in the contact and interaction of virus on the epithelium. The cell surface SA facilitated viral infection and the desialylation of cell could serve as the preventive tool for SARS-CoV-2 pandemic [57]. In particular, α2-6-linked SA compounds have obvious inhibitory effect on SARS-CoV-2 infection and can reduce the expressions of viral RNA and SARS-CoV-2 N protein [58]. SA derivatives have been found to be useful in anti-influenza drug development [59]. Zanamivir and oseltamivir are NEU inhibitors for the prevention and treatment of influenza viruses [60]. EBN extract plays an effective role against viruses. Notably, Neu5Ac, the major molecular specie of SA in EBN, could prevent influenza A virus by inhibiting autophagy process and decreasing the expressions of Rab5 and RhoA [2]. Ovomucin is a glycosylated protein containing SA and has strong inhibitory effects on various pathogens. SA was the important recognition site involved in the binding of ovomucin and hemagglutinin of influenza viruses and exerted anti-influenza virus activity [61]. These antiviral properties are related to the sialylated glycoconjugate. Furthermore, there are pieces of evidence about the anti-viral effect of SA residues on rotavirus [62], human parainfluenza virus [63] and other viruses [64,65,66]. Based on these studies, it provides a reference for further study on the molecular mechanism of SA against virus.

### 3.3. Anti-Inflammation

Inflammation is a physiological defense response against detrimental stimuli, like injurious pathogens, chemical irritation, and allergens. In the inflammatory process, large amounts of signaling molecules are released, such as interleukin-1β (IL-1β), interleukin-6 (IL-6), inducible nitric oxide synthase (iNOS), cyclooxygenase-2 (COX-2), and tumor necrosis factor-α (TNF-α) [67]. The inflammation is associated with some inflammatory diseases, including diabetes, high blood pressure, atherosclerosis, and obesity [68,69]. The SA on the surface of leukocytes can be specifically bound to the E-selectin secreted by vascular endothelial cells, which contributes to transfer leukocytes to inflammatory tissues. SA and its derivatives, as anti-inflammatory agents, can effectively ameliorate the accumulation of leukocytes by blocking the combination of leukocytes and vascular endothelial cells.

The anti-inflammatory effect of SA also was reported for high fat diet-induced inflammation in rats. After an intake of simvastatin (10 mg/kg/day) or Neu5Ac (50 and 400 mg/kg/day) for 12 weeks, the productions of pro-inflammatory substance such as IL-6, TNF-α, C-reactive protein and nuclear factor-kappa B (NF-κB) were lowered significantly. Neu5Ac and simvastatin may have similar potentials for the attenuation of inflammation. Interestingly, the worsened liver and kidney functions existed in the simvastatin groups, and Neu5Ac could protect the regeneration of hepatocytes [44]. Alternatively, as an abiotic SA, SA-2 (with N-butyryl moiety at C-5) markedly prevented NF-κB and mitogen-activated protein kinase (MAPK) signaling in lipopolysaccharide (LPS)-induced RAW 264.7 cells. It lowered the phosphorylation of IκBα, extracellular signal-regulated kinase (ERK)1/2, p38, c-Jun N-terminal kinase (JNK), and the secretion of IL-6 [45]. Siglecs are SA-binding immunoglobulin-type lectins, and soluble Siglec-9 has anti-inflammatory effects. The addition of soluble Siglec-9 inhibited TNF-α-induced inflammatory response in the intestinal epithelial cells, and in the LPS-induced RAW 264.7 cell model, which was worked by inhibiting NF-κB pathway and reducing gene expressions of inflammatory factors (IL-8 and TNF-α). In addition, soluble Siglec-9 significantly reduced intestinal inflammatory symptoms in mice with acute and chronic colitis [46]. Ganglioside GD1a, one of SA-containing glycosphingolipids, could repress the productions of pro-inflammatory cytokines (IL-1β, TNF-α, COX-2 and iNOS), nitric oxide (NO) and PGE2 in the LPS-induced RAW264.7 macrophages. Protective effect of ganglioside GD1a was associated with toll-like receptor 4 (TLR4)/MAPK/NF-κB signaling pathway [47]. On the other hand, our previous study verified the anti-inflammatory effect of SA on ulcerative colitis, which could significantly inhibit MAPK-NF-κB/AP-1 signaling pathway and regulated apoptosis-related protein expressions [48]. Careena et al. [49] also reported that EBN reduced the productions of TNF-α, IL-1β, and IL-6 in an endotoxin-induced neuroinflammation model of Wistar rats, which also indicated the anti-inflammatory effect of SA. These results suggest that SA can regulate inflammation-related signaling pathways and exert its anti-inflammatory effect by inhibiting the expressions of inflammatory factors.

### 3.4. Anti-Oxidant Activity

Many diseases, such as neurodegenerative diseases, atherosclerosis and coronary artery disease, are caused by oxidative stress [70,71]. Several studies have shown the effect of SA on antioxidant capacity. SA can act as an active oxygen scavenger to inhibit hydrogen peroxide-induced cell death [72]. In the atherosclerosis model, apolipoprotein E-deficient mice fed with high-fat diet, oxidative stress was notably reduced by Neu5Ac treatment. Neu5Ac markedly increased the expressions of antioxidant proteins (paraoxonase 1 and 2). Neu5Ac supplementation significantly elevated the activities of SOD, catalase (CAT), and glutathione peroxidase (GSH-Px) in mice [50]. In a similar study, Neu5Ac supplementation obviously improved oxidative stress through lowering thiobarbituric acid reactive species (TBARS) and upregulating mRNA levels of antioxidant genes (GSH-Px, glutathione reductase and SOD) [44]. The nuclear factor erythroid 2-related factor 2 (Nrf2) pathway contributes to antioxidant defence of vascular endothelial cells. Glycocalyx SA can effectively against oxidative stress via the modulation of Nrf2 signaling pathway, up-regulating the expressions of heme oxygenase-1 (HO-1), NADPH quinone oxidoreductase 1 (NQO-1) and glutamate cysteine ligase modifier subunit in the HUVEC [51]. It suggests that SA could represent a novel cure for cardiovascular diseases. On the other hand, SA from EBN effectively inhibited the oxidative stress by reducing the productions of reactive oxygen species (ROS) and TBARS [49]. Hyperglycemia-induced oxidative stress is a risk factor for diabetic vascular complications. SA derived from hydrolyzed EBN successfully prevented the high glucose-induced productions of vascular superoxide and ROS; SA reduced the levels of NADPH oxidase 2 and nitrotyrosine, and increased the levels of antioxidant protein (SOD-1) and p-eNOS protein [52]. As mentioned above, SA can modulate oxidative stress-related signaling pathways and expressions of key target genes to exert antioxidant function.

### 3.5. Affecting Tumor

Growing evidence demonstrated that SA was extremely important in the process of tumorigenesis, immune evasion and metastasis [73,74]. Abnormal expression of SA is a typical symbol of tumor cells. β-galactoside α2,6-sialyltransferases is closely related to tumorigenesis and tumor progression. High levels of β-galactoside α2,6-sialyltransferases mediated tumorigenesis and metastasis in hepatocellular carcinoma cells by activating the Wnt/β-catenin pathway [75]. Human epidermal growth factor receptor 2 (HER2) is related to cell proliferation and migration, and its overexpression can promote carcinogenesis. β-galactoside α2,6-sialyltransferases could induce high HER2 α2,6-sialylation, which promoted the proliferation and invasion of tumor cells through protein kinase B (Akt) and ERK signaling pathways [76]. Meanwhile, with the aggravation of the disease, the content of SA was increased gradually, and the content of SA was decreased gradually during the remission period [77,78]. It may provide a novel approach to the diagnosis and therapeutic target of tumors. Ac_5_3F_ax_Neu5Ac, a SA blockade, exhibits tumour immune responses and reduces tumor growth in several tumor models. Intratumoral injections of 30 mg/kg Ac_5_3F_ax_Neu5Ac significantly blocked B16-F10^WT^ tumors growth, and increased the median survival time from 24 days to 47 days. In the 9464D neuroblastoma model, 20 mg/kg injections remarkably prolonged the median survival from 40 days to 58 days, whereas 10 mg/kg Ac_5_3F_ax_Neu5Ac injections markedly led to tumor regression in B16-F10^OVA^ model [79]. SA-cholesterol conjugate was modified on the epirubicin-loaded liposomes surface and it presented the excellent antitumor activity. It improved the delivery of epirubicin to tumor-associated macrophages, and the survival rate of tumour bearing mice was up to 83.3% [80]. Sialidases NEU1 is a crucial modulator of tumor formation. Downregulation of NEU1 promoted the aberrant expression of SA, which was positively related to the bladder tumor progression. NEU1 significantly induced apoptosis and inhibited tumor formation by interfering with Akt signaling pathway [74]. These results suggest that SA can regulate carcinogenesis-related signaling pathways and the related gene expressions to exert its anti-cancer effect.

### 3.6. Immune-Enhancing Ability

An increasing number of evidences point out that SA is capable of regulating immunity. Siglecs act as transmembrane receptors on the immune cells and mediate immune responses. They modulated receptor pathway in pathological conditions, such as inflammation and cancer [81]. Siglecs represent the attractive opportunity for future treatments of autoimmune diseases. Allergic disease is a serious public health problem in the world. Sialylation of immunoglobulin E (IgE) is the key contributing factor in triggering the allergic cascade. Administering asialylated glycoproteins or removal of SA from IgE attenuated allergic reactions, and the IgE-sialylation could be an important target for allergic intervention [82]. Siglecs are the essential recognition receptor involved in allergic immune responses. Carbohydrate (such as SA)-conjugated allergens binding to Siglecs on immune cells were known to induce suppressive responses [83]. This provides a promising strategy for improving allergen immunotherapy security and effectiveness. SA has shown great potential in the development of nutrition-based immunomodulatory therapies for food allergies, including antiallergic foods. Sialylated polysaccharides have anti-allergic effects, especially on food allergy [84]. Additionally, SA plays a pivotal role in fetal-maternal immune homeostasis during pregnancy. It protected fetal extraembryonic tissue against maternal complement attack [85]. SA also exhibits the immune-enhancing property via antiviral ability. Coronaviruses (CoVs) have biological diversity and pathogenic potential, and they are pathogens in intestinal and respiratory infections. Sialylation pattern was important in glycan-dependent CoVs infection, and SA-Siglec axis was relevant to the immune-based pathogenesis. SA could be as adjunctive drug for the protection and immunotherapeutic strategies against CoVs infections [86]. These investigations deepen the study of SA in the immunization field.

### 3.7. Other Functions

SA also has some other biological functions, for example, it was found that SA can greatly improve the absorption of nutrients, because SA with the strong negative charge is easy to combine with positive charge of Ca^2+^, Mg^2+^, vitamin B12, etc., and promote their transports and absorptions [87]. SA plays an important role in the sprouting and plasticity of neurons, the growth of nerve cells and the regulation of synaptic signal. SA plays a central part in the fields of neuroscience and neurology [88]. SA-modified microsphere, nanoparticles and liposomal, as a promising drug formulation, exhibited the ideal targeted characteristic and could become a hot topic in the treatment of the disease [89,90,91]. SA plays an important role in the homeostasis of cells and organisms and is essential to the developmental biology of the lung [92]. Damaged mitochondria are known to interfere with neuron-based activity. SA could increase mitochondrial membrane potential and alleviated mitochondrial dysfunction [54]. In children with autism, SA levels were negatively associated with autistic-like behaviors, especially stereotyping and hyperactivity [93]. Sialylated IgG has a potential application in alleviating pathogenic autoimmunity. IgG Fc sialylation can up-regulate the inhibitory FcγRIIb of immune cells, decrease the affinity of IgG to complement protein, and thus alleviate the symptoms of autoimmune diseases [94]. This evidence suggests that SA-based disease diagnosis and treatment strategies have made some progress. Tyrosinase is the key enzyme for browning. EBN can strongly inhibit tyrosinase activity in B16 cells and decreases melanin production, and its mechanism is closely related to the release of Neu5Ac by EBN [21]. In the same study, Neu5Ac also exhibited osteogenic activity. MG-63 cells treated with 10–1000 μg/mL EBN digesta for 9 days, alkaline phosphatase activity was increased by 30–40%, and the expression of runt-related transcription factor 2 was also significantly elevated in osteoblasts [21]. In the future research, SA may also exert novel functions, but the molecular mechanisms of these functions need to be further studied.

## 4. Broad Impact of SA on the Gut Microbiome and Function

A large number of investigations indicate the impact of microbiota-host interplay on health implications. Dietary intervention exerts an impact on various physiological processes by modifying the gut microbiota ecosystem. Emerging evidence suggested the key role of SA in the regulation of gut microbiome and host health, although the action mechanism needs to be further clarified [9,95]. Here, the association targeting SA on the modulation of gut microbiome is elucidated.

### 4.1. Effect of SA on the Alterations of Microflora and Host Health

HMOs were the major bioactive nutrients to support brain and cognitive development in human milk, and they were benefit for the infection protection, gut health and maintain homeostasis [96,97]. HMOs, in particularly, and sialylated HMOs, which contained one or more SA residues, were essential bioactive components for the growth of probiotic bacteria in the gut. 3′SL and 6′SL were the predominant sialylated HMOs and they increased the relative abundance of *Bifidobacterium longum* JCM7007, 7009, 7010, 7011, 1272, 11347, ATCC15708, *B. thetaiotaomicron* ATCC29148, and *Bacteroides vulgatus* ATCC8482 [98]. 6′SL-treated piglets increased the prevalence of genera *Faecalibacterium* and *Ruminococcus* (phylum Firmicutes), species *Collinsella aerofaciens* (phylum Actinobacteria), and genus *Prevotella* (phylum Bacteroidetes). Meanwhile, the abundance of families Enterobacteriaceae and Enterococcaceae (phylum Proteobacteria), family Lachnospiraceae and order Lactobacillales (phylum Firmicutes) were reduced in piglets [99]. Supplemented with 3′SL or 6′SL significantly enhanced the bacterial clearance in *Pseudomonas aeruginosa* K-infected mice, which could be applied for the treatment of bacterial and viral infections [100]. Necrotising enterocolitis (NEC) is an inflammatory intestinal disease in preterm infants. The relative proportion of *Bifidobacterium longum* was decreased and the relative abundance of *Enterobacter cloacae* was increased in the infants with NEC. It could be associated with low HMO disialyllacto-N-tetraose [101]. What’s more, the NEC was relieved, along with the inhibition of TLR4 pathway [55]. It means the modulatory effects of gut microbiota by sialyllactose may contribute to TLR4 signaling pathway suppression in NEC. From these evidences, dietary sialyllactose is involved in the regulation of gut microbiome, potentially making a connection to the host health.

In another study, mice fed diets with 3′SL or 6′SL for two weeks relieved anxiety-like behavior resulting from stress. It was further shown that dietary sialyllactose abolished stressor-induced shifts to community structure of colonic microbiota [102]. In view of these results, the impacts of sialyllactose on the modification of gut microbiota may diminish anxiety-like behavior. Mice intragastrically administered with SA for a week altered the gut pathological state and changed notably the bacterial community. At the phylum level, SA notably improved the abundance of Firmicutes, Actinobacteriota, Gemmatimonadetes and Chlorflexi. At the species level, *Staphylococcu lentus*, *Corynebacterium stationis*, *Jeotgalibaca* sp. PTS2502, *Ignatzschineria indica*, *Sporosarcina pasteurii*, *Corynebacterium urealyticum*, *Facklamia tabacinasalis*, *Sporosarcina* sp. HW10C2, *Oblitimonas alkaliphila*, *Erysipelatoclostridium ramosum*, *Blautia* sp. YL58, *Bacteroids thetaiotaomicron*, *Morganella morganii*, and *Clostridioides difficile* were markedly changed by SA [103]. SA metabolism plays a major part in the maintaining the microbiota. In DSS-induced colitis mice, the abundance of *Escherichia coli* was markedly increased, while low contents of intestinal α-2,3-linked SA could inhibit the growth of *Escherichia coli* in mice [104]. It revealed regulation of SA catabolism could prevent the intestinal inflammation characterized by *Escherichia coli* dysbiosis. SA can also significantly alter the microbial community of piglets. It enhanced the presence of *Prevotella* and *Lactobacillus* species, and decreased the proportion of genera *Escherichia*/*Shigella*, *Eubacterium* and *Ruminococcus* [105]. There was a positive relation between breast milk Neu5Ac concentrations and low obesity risk of infants. Besides, breast milk Neu5Ac modulated the infant gut microflora and bile acid metabolism. *Parabacteroides* may be associated with breast milk Neu5Ac levels. The gut microbiota may be a mediator between Neu5Ac and the growth of infants [8]. A study found that Neu5Gc from red meat induced changes in gut microorganisms (especially *Bacteroides* and *Clostridium*), and the low incidence of some inflammatory disorders was owed to Neu5Gc release from red meat [106]. Host *tp53* mutations is crucial to the development of colitis-associated colorectal cancer. Zebrafish *tp53*-mutant larvae caused the elevated inflammation and dysregulation of SA metabolism. What’s more, *Aeromonas* spp. was aberrantly enriched in *tp53*-mutants. Disturbed SA metabolism through a *tp53* mutation may elicit the gut dysbiosis and inflammation [107]. Sialylated IgG is a sort of glycoproteins that modulate the intestinal microorganisms. The proportion of *Bifidobacterium* was increased obviously by sialylated IgG [108]. In a similar study, sialylated IgG has the prebiotic effect in the inflammatory bowel disease (IBD). It significantly enhanced the growth of *Romboutsia*, *Prevotella*, *Akkermansia*, *Bifidobacterium*, *Actinomyces*, *Atopobium* and *Citrobacter*, with low relative abundance in IBD fecal samples. Also, Sialylated IgG promoted the gene expression of *Bifidobacterium bifidum* to regulate the gut microbial composition of IBD patients [109]. All the above studies are further proof of the involvement of microbial ecosystem in SA-mediated protection.

Linear growth faltering is one of serious health problems in undernourished children. A study was conducted to examine the interaction among intestinal gut microbes, HMOs and bone biology. The bone growth was markedly increased in sialylated milk oligosaccharides-fed mice [110]. SA or sialylated glycans can significantly impact the gut microbial composition. In the preclinical model, it showed a microbiota-dependent correlation between sialylated bovine milk oligosaccharides and growth improvement [111]. Song et al. [112] explored the effects of sialylated lactuloses administration on the intestinal microbiota in mice. At the phylum level, the relative proportion of Firmicutes was increased and the proportion of *Bacteroidetes* was decreased. At the family level, the relative abundance of Bacteroidales S24-7 group and Helicobacteraceae was decreased and the abundance of Lactobacillaceae was enhanced. At the same time, the diversity indices of Chao, Shannon and Simpson were higher compared to the normal control group. Specially, the regulation of the sialylated lactulose on intestinal microecology were accompanied by the improvement of immunity. Comparing with control group, the IgA and IgM contents of spleen were raised by 49.05% and 47.25%, and the phagocytic percentage and phagocytic index were greatly higher in KDN-α2,3-lactulose treated group [53,113]. Prebiotics can be acted as the bioactive components and impart various physiological benefits on human health. They can increase nutrients absorption and gut microflora, as well as inhibited the population of pathogens [114]. Galacto-oligosaccharides (GOS), the crucial composition of oligosaccharide-SA, can also be consumed as prebiotics targeting the increment in concentrations of beneficial *Bifidobacterium* directly, and positively promote the anti-inflammatory cytokine (IL-10) expression or diminish the pro-inflammatory factor (IL-1β) level [115,116]. Collectively, SA could exert health benefits through the modulation of gut microbiome structure, which may further provoke changes of gene expressions.

### 4.2. Effect of SA Metabolism

The metabolites not only act as the key source of nutrition for the host, but also are responsible for functions. Numerous studies indicated that SA could be metabolized into microbial metabolites by intestinal microbiota and these metabolites were pivotal players in subsequent metabolic reactions [9]. Short-chain fatty acids (SCFAs) were the main products of microbial metabolism and these fatty acids assisted in gut barrier function, reducing inflammation, keeping the intestinal homeostasis, and pathogen control, accompanied by the regulation of gene expression [117,118,119]. 3′SL and 6′SL increased the productions of SCFAs and lactate by regulation of *Bifidobacterium* [120]. 3′SL and 6′SL were fermented by individual *Bifidobacterium longum*-dominant infant fecal microbiota in vitro. The relative abundance of *Bifidobacterium longum* were increased, and acetate and lactate were the essential metabolites [121]. In terms of bone development, sialylated milk oligosaccharides increased the levels of succinate and improved the state of stunting in gnotobiotic mice colonized with the stunted infant’s intestinal microbiota [110]. Besides, breastmilk components are linked to fecal SCFA concentrations, and the differences in metabolites partially affect the weight gain of infants. Fecal branched SCFA concentrations were positively correlated with sialylated-HMOs, and the concentration of fecal butyrate remained higher in the normal weight gain of infants aged 9 months, as compared with high weight gain group [122]. It suggests that sialylation could improve the infant growth by modulating microbial metabolites. 6′SL-deficient maternal milk markedly impacted tryptophan metabolism, which was related to deficits in behavioral flexibility and cognitive capabilities [37]. Breast milk Neu5Ac was highly correlated with bile acid metabolism in infants. Taurochenodeoxycholic acid 3-sulfate and taurodeoxycholic acid 3-sulfate were associated with low infant obesity risk and breast milk Neu5Ac [8]. SA supplementation is also implicated in the tumor development. SA treatment enhanced the contents of key metabolites, such as UDP-GlcNAc and CMP-Neu5Ac, and mediated energy depletion in nutrient-deprived cancer cells [123]. This finding supports that the metabolism of SA plays a vital part in cancer progression and metastasis. The SA level was a potential predictor for cancers. The elevated SA level was presented in some types of cancers [124,125]. Several studies demonstrated that the elevation of serum SA level could be involved in the pathological process of disorders. Compared to the control, a higher level of SA in plasma and increased oxidative stress parameters were found in Alzheimer’s disease [126]. High contents of plasma total SA could be as a risk factor for cardiovascular disease [127]. During the progression of coronary artery diseases, the level of Neu5Ac was elevated. The increase of Neu5Ac triggered myocardial injury by activating Rho/ROCK-JNK/ERK pathway [128]. Numerous investigations showed that SA metabolism is relevant to occurrence of atherosclerosis [35]. The desialylated low-density lipoprotein was found in patients with atherosclerosis [129]. SA content is also related to insulin resistance. A higher concentration of serum total SA was found in patients with type 2 diabetes [130]. The aberrant sialylation of glycoconjugate and generation of SA were associated with enzymes in SA metabolism, and the pharmacological intervention of these enzymes might be a promising treatment of diseases. These researches demonstrate convincingly that SA metabolism is crucial for eliciting these functions.

The role of HMOs on the host may be interrelated to gene-microbe interactions by microbial metabolites. Previous studies demonstrated that sialylated HMOs prevented NEC via the regulation of gut microbiome and TLR4 pathway repression [55,101]. Subsequent research was performed to examine the effects of sialyllactose on microbiota composition and SCFA production profiles in the epithelial barrier functioning. Sialyllactose could distinctly support epithelial barrier functioning and alter the production of acetate, propionate, lactate, and butyrate by modulating microbiota composition [131]. Similarly, 28 days of 5 g/kg sialyllactose administration was found to elevate the levels of microbial metabolites, including acetic acid, propanoic acid, and butyric acid, and the proportion of *Lactobacillus*, *Bifidobacterium*, and *Bacillus*, while it alleviated inflammation and intestinal injury by suppressing concentrations of inflammatory cytokine (TNF-α, IL-1β, and IL-6), and increasing the expressions of tight junction proteins and fatty acid transport protein-4 in *Escherichia coli*-challenged weaned pigs [132]. Sialylated IgG has the protective role in the prevention of IBD. It caused a marked increase in the contents of SCFAs in IBD samples by in vitro fermentation. Additionally, the metabolic pathway results showed that the functional genes associated with hydrolysis, utilization and absorption of carbohydrates were upregulated by sialylated IgG [109]. In addition, GOS consumption exhibited a rise in the productions of lactic acid and acetic acid, accompanied by the regulatory gene expressions (IL-10 and IL-8) [115,116]. Taking all these investigations into consideration, we might conclude that interactions among intestinal microbes, metabolites and gene expressions matter for the health-promoting effects.

## 5. Conclusions and Prospects

SA is essential for brain development, improvement of learning and memory, anti-virus activity, anti-oxidation and anti-inflammatory function, anti-tumor effect, immunomodulation, disease diagnosis and monitoring. Most studies focus on the biological functions of SA, but the underlying mechanisms are still poorly understood.

**Figure 5:**
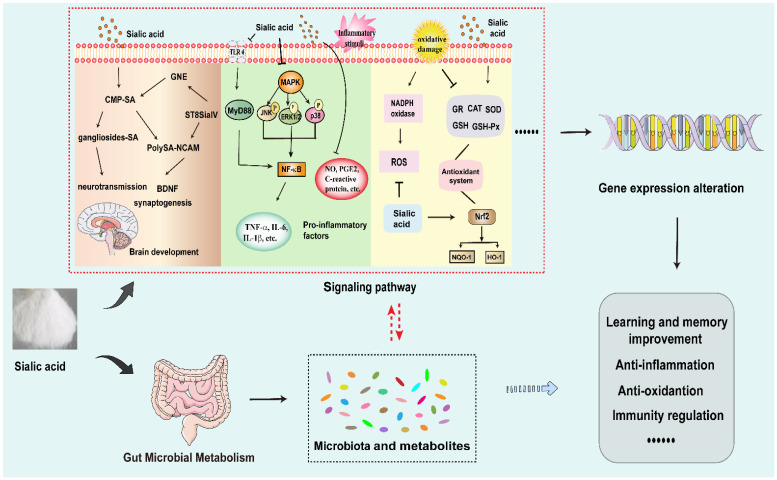
Possible action mechanisms of sialic acid mediated by the signaling pathway and gut environment. Sialic acid (SA) exhibits various biological roles, such as brave development and learning improvement, anti-inflammatory effect, anti-oxidant activity, immune-enhancing ability, and so on. It is worked by the regulation of several signaling pathways, including GNE (UDP-N-acetyl-glucosamine-2-epimerase/N-acetylmannosamine kinase), polysialyltransferase-2,8-sialyltransferase IV, polysialic acid (PolySA), brain-derived neurotrophic factor (BDNF), mitogen-activated protein kinase (MAPK) signal, nuclear factor-kappa B (NF-κB) pathway, toll-like receptor 4 (TLR4) pathway, nuclear factor erythroid 2-related factor 2 (Nrf2) pathway, etc. Meanwhile, SA shows significant functional properties in the host-microbe interactions. It modulates gut microbiota and microbial metabolites. There may be a possible relationship between gut microbiome and gene expression.

SA can reshape the gut microbiota, leading to changes in metabolic products. The metabolites can regulate relevant signaling pathways and the expressions of some key target genes to exert their biological effects [119,133]. But which metabolites are the key regulatory components in the different biological effects? What proteins do the metabolites bind to when it enters the cell? How to interfere with intracellular signaling pathways and transcript factors (see Figure 5)? These issues need to be further studied.

Unfortunately, there is no clinical experimental study of SA. Because of species differences, the biological effects found in animal experiments are not guaranteed to be the same as those found in humans. Only clinical experimental research can promote the direct application of SA, and this field should be an important research direction in the future.

SA has no toxicity and can bring a lot of health benefits. It has great application potential in functional food, medicine, cosmetics and other fields. In-depth study on the biological functions and molecular mechanisms of SA will provide a scientific theoretical basis for its development.

## Figures and Tables

**Table 1:** Studies on biological functions and the molecular mechanisms of sialic acid.

Function	Type	Model	Results	Reference
Learning and memory improvement	Neu5Ac 6′-Sialyllactose	Rat	Memory and long-term potentiation ↑; polysialylated-neural cell adhesion molecule expression ↑.	[36]
	Casein glycomacropeptide	Piglet	Brain development ↑; learning-associated genes (ST8SiaIV and GNE) ↑.	[38]
	6′-Sialyllactose	Mice	Cognitive development ↑; genes mediating neuronal patterning ↑.	[37]
	Sialyllactose	Pig	Brain development ↑; genes encoding SA metabolism, myelination, and ganglioside biosynthesis ↑.	[39]
	Edible bird’s nest	Mice	Cognitive and neurological development ↑; GNE, ST8SiaIV, SLC17A5, and BDNF in the generation mice ↑.	[42]
	Edible bird’s nest	Mice	Learning and memory performances in the offspring ↑; BDNF, SOD and ChAT ↑; MDA and AChE ↓.	[43]
Anti-inflammation	Neu5Ac	High fat diet-fed rats	Inflammation ↓; IL-6, TNF-α, C-reactive protein and NF-κB ↓.	[44]
	Abiotic SA	LPS-stimulated RAW 264.7 cells	Inflammation ↓; NF-κB and MAPK signaling ↓.	[45]
	Soluble Siglec-9	TNF-α-stimulated COLO 205 human IECs and LPS-induced RAW 264.7 cells; colitis mice	NF-κB in COLO 205 human IECs and RAW 264.7 cells ↓; intestinal inflammation in colitis mice ↓.	[46]
	Ganglioside GD1a	LPS-induced RAW 264.7 cells	Inflammation ↓; pro-inflammatory cytokines, NO and PGE2 ↓, TLR4/MAPK/NF-κB ↓.	[47]
	Neu5Ac	Colitis mice; LPS-stimulated RAW 264.7 cells	Inflammation ↓; pro-inflammatory factors ↓; MAPK-NF-κB/AP-1↓.	[48]
	Edible bird’s nest extract	LPS-induced rats	Neuro-inflammation ↓; TNF-α, IL-1β, and IL-6 ↓.	[49]
Anti-oxidant function	Neu5Ac	High fat diet-fed apolipoprotein E-deficient mice	Antioxidant enzymes activity ↑, expressions of antioxidant protein (paraoxonase 1 and 2) ↑.	[50]
	Neu5Ac	High fat diet-fed rats	GSH-Px, glutathione reductase, SOD ↑.	[44]
	Glycocalyx SA	Human endothelial cells	Nrf2 signaling ↑.	[51]
	Hydrolyzed edible bird’s nest and SA	Diabetic mice and high glucose-induced HUVEC	NADPH oxidase 2 and nitrotyrosine ↓, anti-oxidant protein (SOD-1) and p-eNOS ↑.	[52]
Immune-enhancing ability	Sialylated lactulose	Mice	IgA and IgM contents of spleen ↑, phagocytic percentage ↑, phagocytic index ↑.	[53]
Osteogenic activity	Edible bird’s nest	MG-63 cell and osteoblast	Alkaline phosphatase expression in MG-63 cells and osteoblast ↑, runt-related transcription factor 2 expression in osteoblast ↑.	[21]
Reduce mitochondrial dysfunction	Edible bird’s nest	SH-SY5Y cell	Mitochondrial dysfunction ↓; expression of active mitochondria ↑.	[54]
Anti-necrotizing enterocolitis	6′-Sialyllactose	Mice and piglet	Necrotizing enterocolitis development ↓; TLR4 signaling ↓.	[55]

“↑” represents “increase”; “↓” represents “decrease”.

## Data Availability

Data is contained within the article.

## Abbreviations

3′SL	3′sialylactose
6′SL	6′sialylactose
Acetyl-CoA	acetyl coenzyme A
AChE	acetylcholinesterase
Akt	protein kinase B
BDNF	brain-derived neurotrophic factor
CAT	catalase
ChAT	choline acetyltransferase
CMAH	cytidine monophosphate N-acetylneuraminic acid hydroxylase
CMAS	cytosine 5′-monophosphate N-acetylneuraminate synthetase
CMP-Neu5Ac	cytidine 5′-monophosphate-N-acetylneuraminic acid
CoVs	coronaviruses
COX-2	cyclooxygenase-2
EBN	edible bird’s nest
ERK	extracellular signal-regulated kinase
GNE	UDP-N-acetlyl-glucosamine-2-epimerase
GOS	galacto-oligosaccharides
GSH-Px	glutathione peroxidase
HER2	human epidermal growth factor receptor 2
HMOs	human milk oligosaccharides
HO-1	heme oxygenase-1
IBD	inflammatory bowel disease
IgE	immunoglobulin E
IL-1β	interleukin-1β
IL-6	interleukin-6
iNOS	inducible nitric oxide synthase
JNK	c-Jun N-Terminal kinase
KDN	deaminated neuraminic acid
LPS	lipopolysaccharide
ManNAc	N-acetylmannosamine
ManNAc-6-P	ManNAc-6-phosphate
MAPK	mitogen-activated protein kinase
MDA	malondialdehyde
NANP	N-acetylneuraminate-9-phosphate phosphatase
NAL	N-acetylneuraminate lyase
NANS	N-acetylneuraminate-9-phosphate synthase
NCAM	neural cell adhesion molecule
NEC	necrotising enterocolitis
Neu	neuraminic acid
NEU	neuraminidase
Neu5Ac	N-acetylneuraminic acid
Neu5Gc	N-glycolylneuraminic acid
NeuAc-9-P	N-acetylneuraminic-9-phosphate
NF-κB	nuclear factor-kappa B
NO	nitric oxide
NQO-1	NADPH quinone oxidoreductase 1
Nrf2	nuclear factor erythroid 2-related factor 2
PEP	phosphoenolpyruvate
PolySA	polysialic acid
ROS	reactive oxygen species
SA	sialic acid
SARS-CoV-2	severe acute respiratory syndrome coronavirus-2
SCFAs	short-chain fatty acids
SLC17A5	solute carrier family 17 member 5
SOD	superoxide dismutase
ST8SiaIV	α-2,8-sialyltransferase IV
TBARS	thiobarbituric acid reactive species
TLR4	toll-like receptor 4
TNF-α	tumor necrosis factor-α
UDP-GlcNAc	uridine diphosphate-N-acetylglucosamine
